# Introduction to the Special Issue: *Dental Chemistry of Calcium Phosphates*

**DOI:** 10.6028/jres.115.014

**Published:** 2010-08-01

**Authors:** Clifton M. Carey

Dear Reader,

This special issue of the Journal of Research of the National Institute of Standards and Technology (NIST) highlights the recent research on calcium-phosphate (Ca-PO_4_) chemistry that is being conducted under the guidance and leadership of Dr. Laurence Chow at the American Dental Association Foundation’s Paffenbarger Research Center (PRC). Dr. Chow is the Assistant Director of the PRC and Chief Research Scientist of the Dental Chemistry group. He has been a part of the Ca-PO4 research team since his arrival in 1969. The papers presented here represent the latest research on Ca-PO_4_ chemistry as applied to medical and dental research, and describe the development of Ca-PO_4_-based bone grafting materials, dental applications such as root canal fillers, nano Ca-PO_4_ and nano CaF_2_ materials for dental remineralization.

After 41 years with the American Dental Association, Dr. Chow has received numerous grants and many honors throughout his career, including chairing several sessions at the Gordon Research Conference and receiving the Basic Research in Biological Mineralization Award from the International Association for Dental Research (IADR), an award given out to only 15 distinguished scientists annually. This special issue from NIST’s Journal of Research takes its place alongside these notable accomplishments.

The PRC is the research unit of the American Dental Association Foundation (ADAF) located at the National Institute of Standards and Technology (NIST) in Gaithersburg, Maryland. The collaboration between the U.S. Government (through the Department of Commerce) and the ADA started from a request for information about amalgams from the National Bureau of Standards (NBS) in 1919. The NBS was responding to the Department of the Army’s need for dental amalgam purchasing specifications. To accomplish this, the NBS needed dental professionals to collaborate in an effort to establish materials standards for amalgams; they requested that the ADA participate in the endeavor. In 1928 the ADA sent Dr. Norris Taylor as a research associate to the NBS thus beginning a very productive collaboration between the ADA and NBS. The NBS Research Associates program continues today and through this ADA-U.S. Government partnership the dental profession has been significantly changed. Our unique environment, being located at NIST under the U.S. Department of Commerce, provides us with the facilities, resources and expertise to be able to take science from the bench to the patient. Our affiliation with the ADA gives us a direct link to the needs of the practicing community. Our host institute, NIST, provides a framework for standards and measurement needs for the dental and medical industry, and our dedicated staff provides the technical expertise necessary to make it all happen.

PRC scientists have played major roles in the development of modern high-speed dental drills, panoramic x-ray machines, face surfaced dental mirrors, protective tooth sealants, orthodontic bracket bonding materials, tooth-colored composite filling materials, calcium phosphate bone cements and adhesives that bond composites and other filling materials to teeth. The inventions are assigned to the ADAF and licensed to dental and medical material product manufacturers worldwide. The income generated through licensing is exclusively devoted to supporting ADAF research and educational programs.

The following list presents some of the accomplishments that stand out as essential to the profession of dentistry. All of these accomplishments have required the continuous collaboration and support of NBS/NIST and the ADA throughout the Center’s illustrious 82-year history.

- 1920’s & 1930’s – ADA scientists developed standards for dental materials in collaboration with NBS and industry scientists
- 1940’s – ADA scientists at NBS initiated what would become the ADA Seal Program, a science-based standard that the ADA uses to evaluate a dental product’s safety and effectiveness
- 1950’s – ADA scientists in collaboration with NBS scientists invented the High Speed Handpiece
- 1960’s – ADA scientists at NBS invented the Panoramic X-Ray
- 1970’s – ADAF scientists at NBS introduced Dental Composites to industry
- 1980’s – ADAF scientists at NBS developed advanced Dentin Adhesives and Fluoride therapies
- 1990’s – PRC ADAF scientists at NIST introduced Bone Cements to industry
- 2000’s – PRC ADAF scientists at NIST are currently working on a range of new materials that produce enamel/dentin regeneration and advanced products for the prevention of dental decay.

The research reported in these papers was supported by an annual grant from the American Dental Association to the ADA Foundation; by in-kind contributions from the National Institute of Standards and Technology (NIST), where the Paffenbarger Research Center is located; by research grants from the National Institutes of Health, through the National Institute of Dental and Craniofacial Research (NIDCR); and by corporate grants and contracts.

